# Postpartum Hemorrhage and Post-Traumatic Stress Disorder : The Role of Trait Anxiety

**DOI:** 10.1192/j.eurpsy.2026.12000

**Published:** 2026-07-31

**Authors:** N. Smaoui, A. Jedidi, N. Charfi, M. Maalej, H. Hkim, R. Feki, I. Gassara, M. Maalej, S. Omri, L. Zouari

**Affiliations:** 1 Psychiatry C; 2 Gynecology-Obstetrics Department, Hedi Chaker Hospital, Sfax, Tunisia

## Abstract

**Introduction:**

Severe postpartum hemorrhage (PPH) is not only a life-threatening obstetric complication but also a potential psychological trauma. While the link between PPH and post-traumatic stress disorder (PTSD) is increasingly recognized, the role of individual vulnerability factors such as trait anxiety remains less well explored.

**Objectives:**

The objectives of this study were to evaluate post-traumatic stress disorder (PTSD) and trait anxiety in women who experienced severe postpartum hemorrhage (PPH), and to determine the relationship between them.

**Methods:**

A cross-sectional, descriptive, and analytical study was conducted between January and December 2024 among 49 participants who presented with severe PPH. These women had delivered between 2017 and 2023 at the Gynecology-Obstetrics Department of Hedi Chaker University Hospital in Sfax.

PTSD was assessed using the *Post-Traumatic Stress Disorder Checklist for DSM-5* (PCL-5), with a diagnostic threshold set at a total score ≥ 31. Trait anxiety was assessed using the *Spielberger Trait Anxiety Inventory* (STAI-Y).

Statistical analysis was performed using SPSS-26 software.

**Results:**

The mean age of participants at the time of delivery complicated by PPH was 31.16 ± 5.86 years (min = 19, max = 47), and 34.16 ± 6.03 years (min = 23, max = 49) at the time of the interview.

The mean STAI-Y score was 49.39 ± 11.395 (min = 32, max = 72). Trait anxiety was present in 51% of participants.

The mean PCL-5 score was 31.81 ± 18.54 (min = 4, max = 71), with a PTSD prevalence of 44.9% (n=22).

Mean scores for the PCL-5 dimensions were 7.98 ± 5.59 for re-experiencing (min = 0, max = 18), 3.78 ± 2.53 for avoidance (min = 0, max = 8), 9.84 ± 8.53 for negative alterations in cognition and mood (min = 0, max = 28), and 9.61 ± 4.83 for hyperarousal (min = 0, max = 22).

A significant correlation was observed between the PCL-5 and STAI-Y scores, with a Pearson coefficient r = 0.564 (p < 0.01), indicating a positive association between PTSD and trait anxiety. In multivariate analysis, trait anxiety (STAI-Y) (OR = 5.14; 95% CI 1.52–17.38; p = 0.008) remained a significant independent predictor of PTSD.

**Conclusions:**

Severe PPH is a highly traumatic event. Chronic anxiety appears to be an important risk factor for PTSD, underscoring the need for targeted preventive and therapeutic strategies.

**Disclosure of Interest:**

None Declared

